# HGF potentiates extracellular matrix-driven migration of human myoblasts: involvement of matrix metalloproteinases and MAPK/ERK pathway

**DOI:** 10.1186/s13395-017-0138-6

**Published:** 2017-10-10

**Authors:** Mariela Natacha González, Wallace de Mello, Gillian S. Butler-Browne, Suse Dayse Silva-Barbosa, Vincent Mouly, Wilson Savino, Ingo Riederer

**Affiliations:** 1 Laboratory on Thymus Research, Oswaldo Cruz Institute, Oswaldo Cruz Foundation, Av. Brasil 4365, Manguinhos, Rio de Janeiro, 21045-900 Brazil; 2 Brazilian National Institute of Science and Technology on Neuroimmunomodulation (INCT-NIM), Av. Brasil 4365, Manguinhos, 21045-900 Rio de Janeiro, Brasil; 3 Sorbonne Universités, Université Pierre et Marie Curie, INSERM UMRS974, CNRS FRE3617, Center for Research in Myology, 47 Boulevard de l’hôpital, 75013 Paris, France; 4 Department of Clinical Research, National Cancer Institute (INCA), Rio de Janeiro, Brazil

**Keywords:** Myoblast transplantation, HGF, Migration, Laminin, Fibronectin, Matrix metalloproteinases, MAPK/ERK

## Abstract

**Background:**

The hepatocyte growth factor (HGF) is required for the activation of muscle progenitor cells called satellite cells (SC), plays a role in the migration of proliferating SC (myoblasts), and is present as a soluble factor during muscle regeneration, along with extracellular matrix (ECM) molecules. In this study, we aimed at determining whether HGF is able to interact with ECM proteins, particularly laminin 111 and fibronectin, and to modulate human myoblast migration.

**Methods:**

We evaluated the expression of the HGF-receptor c-Met, laminin, and fibronectin receptors by immunoblotting, flow cytometry, or immunofluorescence and used Transwell assays to analyze myoblast migration on laminin 111 and fibronectin in the absence or presence of HGF. Zymography was used to check whether HGF could modulate the production of matrix metalloproteinases by human myoblasts, and the activation of MAPK/ERK pathways was evaluated by immunoblotting.

**Results:**

We demonstrated that human myoblasts express c-Met, together with laminin and fibronectin receptors. We observed that human laminin 111 and fibronectin have a chemotactic effect on myoblast migration, and this was synergistically increased when low doses of HGF were added. We detected an increase in MMP-2 activity in myoblasts treated with HGF. Conversely, MMP-2 inhibition decreased the HGF-associated stimulation of cell migration triggered by laminin or fibronectin. HGF treatment also induced in human myoblasts activation of MAPK/ERK pathways, whose specific inhibition decreased the HGF-associated stimulus of cell migration triggered by laminin 111 or fibronectin.

**Conclusions:**

We demonstrate that HGF induces ERK phosphorylation and MMP production, thus stimulating human myoblast migration on ECM molecules. Conceptually, these data state that the mechanisms involved in the migration of human myoblasts comprise both soluble and insoluble moieties. This should be taken into account to optimize the design of therapeutic cell transplantation strategies by improving the migration of donor cells within the host tissue, a main issue regarding this approach.

**Electronic supplementary material:**

The online version of this article (10.1186/s13395-017-0138-6) contains supplementary material, which is available to authorized users.

## Background

Muscle repair after degeneration caused by trauma or disease is physiologically ensured by proliferating myoblasts, derived from satellite cells (SC), which are the muscular progenitors, located beneath the basal lamina and the sarcolemma of muscle fibers [1]. After proliferation, myoblasts exit cell cycle, differentiate and fuse with other myoblasts to create new fibers, or with damaged fibers, allowing tissue regeneration [2]. The process of muscle regeneration, from SC activation to fiber repair, is orchestrated essentially by members of the Myogenic Regulatory Factors (MRF), such as MyoD and myogenin [3, 4], and occurs in the presence of inflammatory cells, extracellular matrix (ECM) proteins, and soluble factors [5–7].

After activation and exit of the SC niche, myoblasts migrate, so that to reach the damaged fibers or to align to other myoblasts, and create new muscle fibers. Migration is thus an essential step in skeletal muscle regeneration. A gradient of chemokines, cytokines, and growth factors stimulate and guide this migratory process, and ECM components not only serve as an haptotatic substrate to the migrating cells but can also bind, store, and concentrate soluble factors, improving cell motility [8–11]. Moreover, ECM remodeling provoked by proteolytic enzymes, namely matrix metalloproteinases (MMPs), also releases growth factors sequestered by this network, increasing their chemoattractant potential by enzymatic cleavage [12, 13].

Laminin (LN) and fibronectin (FN) are ECM glycoproteins able to stimulate the migration of different cell types [14–18], including myoblasts [19–21]. LN is the major glycoprotein of the basal membrane in different tissues, and the isoforms 211 and 221 surrounds the muscle fibers and the SCs [22]. Although the SC niche does not contain FN, after muscle injury, the expression of this molecule is transiently enhanced, peaking on day 5 [23]. So, while LN isoforms are in contact with quiescent SCs, after activation, myoblasts are surrounded by both LN and FN. Moreover, it has recently been shown that different LN isoforms also have distinct effects on proliferation and differentiation of murine and human myoblasts: while LN-211, which is present on resting muscle fibers, stimulates neither proliferation nor differentiation, LN-521 stimulates both processes in vitro [24].

It has been demonstrated that hepatocyte growth factor (HGF) is a potent muscle stem cell activator, playing an important role in muscle development by promoting the recruitment of myogenic precursors from somite to early muscle areas in the developing embryo [25]. In the mature muscle, HGF is involved in the early activation of quiescent SC before entering into the cell cycle [26]. HGF also plays a role in regulating the different steps of myogenesis, i.e., proliferation, migration, and differentiation, in a paracrine manner [27–29]. HGF is secreted as a 92 kDa biologically inactive single polypeptide chain precursor referred to as pro-HGF, which binds to the ECM through heparin-like glycosaminoglycans [30].

Extracellular proteolytic enzymes, such as urokinase plasminogen activator, thrombin, and matriptase, can cleave pro-HGF into a bioactive disulphide-linked heterodimer consisting of a 60 kDa α-chain and a 32 kDa β-chain [31, 32], which is now able to activate intracellular signaling by binding c-met, a transmembrane tyrosine kinase receptor encoded by the MET proto-oncogene [33]. Interestingly, MMPs are involved in pro-HGF cleavage and activation, when the muscle is injured, exercised, overworked, or mechanically stretched [34, 35].

Previous work has demonstrated that various chemotactic factors, including HGF, can modulate, at least in vitro, the process of myoblast migration across an endothelial barrier [36]. It has also been demonstrated that the association between HGF and an ECM-containing network (matrigel) can improve survival dispersion, proliferation, and differentiation of myogenic cells in a system of ectopic skeletal muscle transplantation [37].

Taken together, these data led us to raise the hypothesis that HGF, associated with ECM, might be involved in the migration of muscle progenitors, consequently being relevant for the efficiency of cell therapy protocols for treatment of muscular diseases. The demonstration that in vitro expanded SC (myoblasts) were able to fuse with recipient fibers when transplanted into mouse muscles [38] rapidly led to clinical trials, mainly to treat Duchenne Muscular Dystrophy (DMD), the most severe form of muscular dystrophies. However, no clinical benefits were observed [39–41]. Despite these initial negative results to treat DMD, clinical trials continued due to the encouraging results obtained in oculopharyngeal muscular dystrophy (OPMD) [42] where the myoblasts transplanted do not need to migrate long distances to achieve clinical benefit. Also, limited migration of the injected cells is a major problem related to myoblasts transplantation in experimental animal models [43–45].

Considering myoblast injection as a potential treatment for at least, some muscular disorders, substantial dispersion inside the muscle will be necessary to improve this approach. LN isoforms could be instrumental in this context, and LN-111 has already been used as a therapeutic agent in different animal models, despite its absence in normal adult muscles [22].

In the present study, we examined the effect of HGF upon ECM-driven migration of human myoblasts. Our data show that low doses of HGF per se do not trigger a migratory response but do potentiate migration of human myoblasts driven by ECM proteins, namely FN and LN-111. This effect seems to involve MMPs and the MAPK/ERK signaling pathway since it can be abrogated by specific inhibitors.

## Methods

### Human myoblast preparation and cell culture conditions

All human myoblast cultures were derived from biopsies obtained anonymously from MyoBank, a tissue bank affiliated to EuroBioBank (authorization from the French ministry no. AC-2013-1868). The primary human myoblasts (CHQ) used in this study were already described. CHQ is an expanded primary culture isolated from quadriceps of a 5-day-old female, with no signs of neuromuscular disease, and used during the first 35 divisions to avoid any bias due to replicative senescence, since their lifespan is 55 divisions [46]. Proliferating cells were maintained in vitro in growth medium composed of DMEM plus medium 199 (supplemented with 50 μg/ml gentamycin and 20% fetal bovine serum [47]. The cells were incubated at 37 °C in a 5% CO_2_atmosphere, and experiments were usually carried out between 20 and 35 population doublings, so largely before replicative senescence. Additionally, we used two immortalized cell clones: LHCN-M2 and CL-25 (derived from the CHQ primary culture) [47]. Immortalized cells were maintained in vitro in same growth medium except it was supplemented with 25 μg/ml fetuin, 0.5 ng/ml basic Fibroblast Growth Factor, 5 ng/ml Epidermal Growth Factor, and 0.2 μg/ml dexamethasone. For the migration assay, human myoblasts were incubated with the “migration medium” (DMEM + 199 medium + 0.5% BSA).

We confirmed the myogenicity of the CHQ human myoblasts primary culture by monitoring the expression of CD56 (N-CAM, neural-cell adhesion molecule) and desmin. In these primary cultures, both molecules are present exclusively in myogenic cells, and in our experiments, only CHQ cultures with more than 75% myogenicity were used. The immortalized cells were 100% myogenic. Differentiation was induced at the high confluence, by switching the medium to DMEM without serum. The human hepatoma cell line HEP-G2 was used as a positive control for c-Met detection [48]. HEP-G2 cells were cultured in DMEM medium supplemented with fetal calf serum 20%, at 37 °C in 5% CO_2_ atmosphere.

### Antibodies and chemicals

The various fluorochrome-labeled or purified antibodies used in the present study are summarized in Table 1.

**Table 1:** Antibodies used in this study

Primary fluorochrome labeled antibodies	Primary purified antibodies	Secondary antibodies
Anti-CD29/PE-Cy5 (Pharmingen/Becton-Dickinson)	Rabbit anti-desmin monoclonal (Millipore)	Goat-anti rabbit Ig/Cy3 (Sigma)
Anti-CD49d/PE (Pharmingen/Becton-Dickinson)	Rabbit anti-CD49g (alpha7-integrin; Abcam)	Goat anti-rabbit Ig/alexa-488 (Sigma)
Anti-CD49e/PE (Pharmingen/Becton-Dickinson)	Rabbit anti-c-met polyclonal (Santa Cruz)	Mouse anti-rabbit Ig, coupled to alkaline phosphatase (Sigma)
Anti-CD49f/PE (Pharmingen/Becton-Dickinson)	Anti-ERK and phosphorylated-ERK rabbit monoclonal (Cell Signaling)	Goat anti-rabbit Ig coupled to peroxidase (SouthernBiotech)
CD56/APC (Pharmingen/Becton-Dickinson)	Anti-actin mouse monoclonal (Millipore)	Goat anti-mouse Ig coupled to peroxidase (SouthernBiotech)

For fibrillar actin detection, we used phalloidin conjugated to alexa-488 (Molecular Probes). Bovine serum albumin (BSA), mouse Laminin-111, human plasma fibronectin, and pork tail-derived gelatin were Sigma-Aldrich products. Recombinant human HGF was obtained from R&D Systems (USA). The specific MMP-2/-9 Inhibitor III [49] was from Calbiochem (USA). The ERK inhibitor UO126 was purchased from Sigma. Lastly, 1 mM p-amino phenylmercuric acetate (APMA, a Sigma product) was applied as an MMP activator, as originally reported [50].

### Cytofluorometry and immunocytochemistry

For flow cytometry, 5 × 10^5^ cells were pre-treated with normal mouse serum and subsequently stained with the target fluorochrome-coupled primary antibodies for 30 min. Cells were washed and fixed with a 1% formaldehyde solution. Samples were analyzed using a FACSCanto II flow cytometer (Becton-Dickinson, San Diego, USA), and histograms were obtained using the FlowJoV10 software.

For immunocytochemistry, cells were fixed using absolute cold ethanol and washed with PBS. Slides were blocked with 10% normal sheep serum for 30 min at room temperature. Primary antibodies were incubated for 1 h in the same conditions and revealed by using either immunofluorescent or enzyme-coupled secondary antibodies.

### Cell adhesion assay

To assess the role of HGF on the adhesive capacity of myoblasts on ECM substrates, Lab-Tek glass slides (Nunc, Thermo Fisher Scientific, USA) were coated for 1 h at 37 °C with 10 μg/ml LN-111, FN, gelatin (GT), or BSA as a negative control. In this assay, 4 × 10^3^ myoblasts were mixed in growth medium, containing or not HGF 10 ng/ml, and were allowed to adhere onto the pre-coated wells for 15 and 30 min and 1 and 2 h. The wells were then washed carefully and fixed during 15 min with paraformaldehyde 2% and staining to F-actin. For F-actin detection, adhered cells were treated with 0.1% saponin, 1% BSA for 15 min at 37 °C and stained with phalloidin alexa-488 for 30 min. Images were acquired using an Axio Imager A2 fluorescence microscope (Carl Zeiss, Germany).

### Cell migration assay

The migratory activity of human myoblasts was assessed in Transwell migration chambers, using 8-μm pore size inserts (Nunc, Roskilde, Denmark). Membrane inserts were coated on both sides with BSA, LN-111 or FN (for 1 h at 37 °C, followed by 1 h of blocking with 0.5% BSA). Myoblasts (10^5^ cells) were plated in the upper chamber in 100 μl of migration medium, and 600 μl of the same medium containing or not HGF at different concentrations were put in the lower chamber, with or without the inhibitor UO126. Cells were allowed to migrate 4 h. In kinetic studies for assaying migration, the cells were allowed to migrate 30 min and 1, 2, and 4 h. Cells that did not migrate through the porous membrane were gently removed from the upper side of the insert. The cells attached to the membrane of the lower chamber were fixed, stained, and washed in a 24-well plate(s) containing 0.5 mL of paraformaldehyde, 0.5 mL of Panotic stain, and 0.5 mL of distilled water, respectively. Inserts were air-dried and counted under the microscope at 400× magnification. Ten random microscopic fields were counted in each insert. Data were expressed as the average of the number of cells/field.

To examine the role of MMPs and MAPK/ERK signaling pathway production on myoblast migration, the MMP-2/-9 Inhibitor III and UO126 were added in both chambers and the cells attached to the lower side after migration were detached by trypsinization and counted.

### Zymography

Detection of MMP/collagenase activity was performed using the gelatin zymography assay. Five hundred thousand human myoblasts were treated with different HGF concentrations and analyzed 72 h later. The supernatants (10 μg) were then collected, mixed with sample buffer in non-reducing conditions, and run on 7.5% polyacrylamide gels co-polymerized with 0.1% gelatin under 40 mA until the dye tracker reached the bottom. After electrophoresis, gels were washed for 1 h in renaturating buffer (2.5% Triton X-100, Tris-HCl 50 mM pH 7,6) at room temperature and subsequently revealed by incubation in developing buffer (5 mM CaCl_2_, 150 mM NaCl, Tris-HCl 50 mM pH 7,6) for at least 16 h. Gels were stained with Coomassie blue for 1 h and destained with acetic acid-methanol-water. MMPs were identified by comparison with the molecular weight standards (BioRad, Hercules, CA, USA). Metalloproteinase activity was visualized as clear bands that were inverted using the Photoshop package. Quantification was performed by densitometry using the ImageJ software.

### Western blotting

Human primary myoblasts (CHQ cells) were incubated with medium alone or with 10 ng/ml HGF in pre-coated plates with BSA, FN or LN-111 and after 15 min post-stimulation, extracts were prepared by lysing cells in alkaline lysis buffer (Tris-HCl 40 mM, SDS 1%). Proteins obtained by this procedure were quantified with the BCA protein kit (Pierce Co., UK). Samples (40 μg) were resolved on 10% polyacrylamide gels, and the proteins were electrotransferred to a nitrocellulose membrane (Hybond, Amersham, Buckinghamshire, UK). The membrane was treated with 5% non-fat milk, 0.1% Tween 20 in PBS for 2 h, and then incubated overnight with the anti-HGF antibody diluted in blocking solution. Membranes were washed three times with PBS and incubated with an alkaline phosphatase-coupled secondary antibody. Bands were visualized using BCIP/NBT solution kit and scanned images were analyzed using the ImageJ software (NIH, Bethesda, USA). For detecting signaling pathway of ERK, we used anti-human ERK and phosphorylated-ERK rabbit monoclonal antibody, and antibody binding was revealed using a peroxidase-coupled goat anti-rabbit Ig serum, and the signal was analyzed using a chemiluminescence detection system (GE-Healthcare). Expression levels were normalized against the α-actin signal.

### Statistical analysis

All results were presented as the mean ± standard error and the statistical differences between groups were detected using the ANOVA test and Newman-Keuls post-test, applying the Prisma 5.0 software. Differences were considered statistically significant when *p* ≤ 0.05.

## Results

### Human myoblasts express HGF, FN and LN receptors

**Fig. 1:**
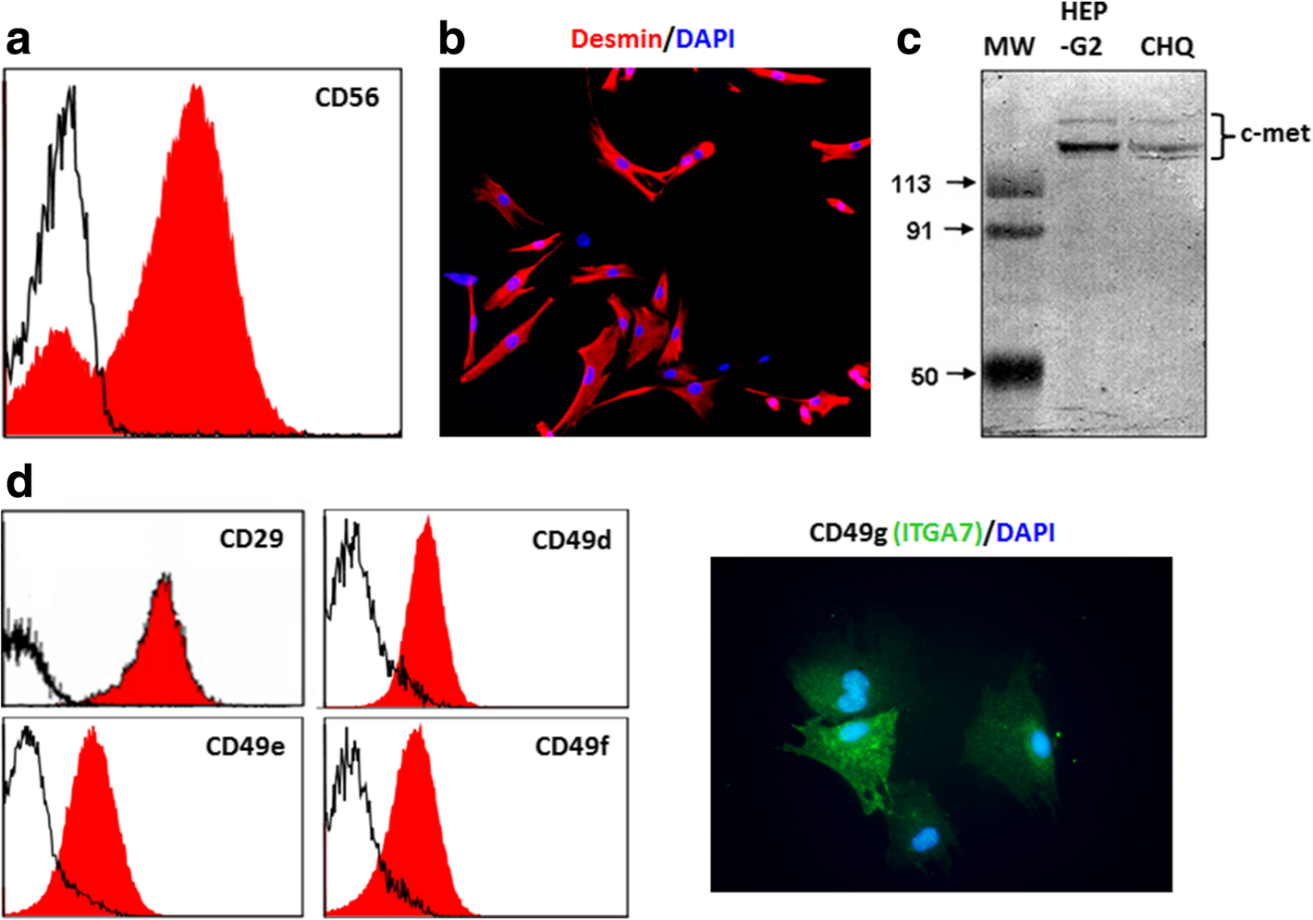
Phenotypic features of the CHQ human myoblast preparation. **a** Culture purity was determined by cytofluorometric detection of CD56, a typical myoblast surface marker. As seen in the FACS profile, 87% of this cell preparation did correspond to myoblasts, since labeling was positive for the anti-CD56 antibody, as compared to the black curves, generated after binding of an unrelated antibody. **b** The myogenicity of the cells was confirmed by desmin expression in immunofluorescence, using an anti-desmin monoclonal antibody. **c** Immunodetection of c-met by western blotting of whole-cell extracts. Hepatoma cell line HEP-G2: positive control. **d** Flow cytometry profiles of the expression of CD29 (β1-integrin chain), CD49d, CD49e (integrin α-chains of the fibronectin receptors VLA4 and VLA5 respectively), and CD49f (integrin α-chain of the laminin receptor VLA6). VLA-7 expression was detected by immunostaining using an anti-CD49g antibody. Data are representative of three independent experiments

Before evaluating whether HGF, together or not with the ECM molecules FN and LN, could trigger a functional response on human myoblasts, we characterized the phenotype of these myogenic precursors focusing on the expression of the corresponding receptors. As seen by CD56 staining, a marker present on myoblasts and absent on fibroblasts (the principal non-myogenic population in these cultures), our cultures presented more than 75% of myogenicity, checked by flow cytometry, and further confirmed by immunohistochemistry with desmin staining (Fig. 1a, b). Their myogenic capacity was also determined by the capacity of these cells to form myotubes positive for myosin heavy chain (MHC), as revealed with the pan-myosin MHC MF20, with more than 70% of fusion (data not shown).

We then investigated in these cells the expression of c-Met, the HGF receptor. As shown in Fig. 1c, human myogenic precursors express c-Met constitutively. We also showed that myoblasts express β1-integrins on their surface, as revealed by positive immunolabeling for CD29 (β1-integrin chain) as well as CD49d, CD49e, CD49f, and CD49g (the integrin α-chains for the FN receptors VLA-4, VLA-5, and the LN receptors VLA-6 and VLA-7, respectively (Fig. 1d, e)). The immortalized cell cultures revealed 100% of myogenicity and exhibited a very similar pattern of integrin receptors when compared to the CHQ cultures (Additional file 1).

### ECM proteins accelerate adhesion in human myogenic precursors

It has been demonstrated that the interaction between HGF and its receptor c-Met regulates muscle cell migration during embryonic development [25] and also in vitro [29]. Given that cell migration requires the regulation of cell adhesion/spreading and the disruption of cell-matrix or cell-cell contact, we first tested the ability of human myoblasts to adhere to substrates containing LN or FN, in the absence or presence of HGF.

**Fig. 2:**
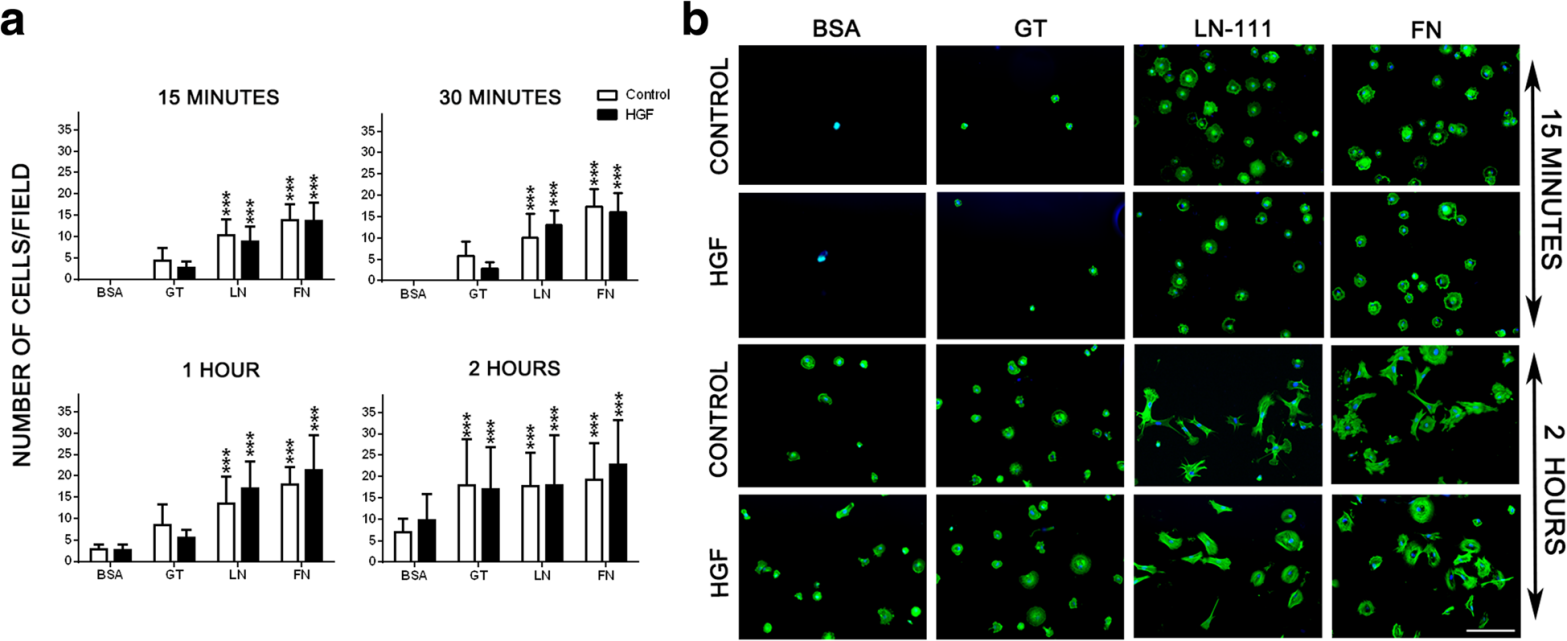
HGF treatment does not affect the adherence of human CHQ myoblast onto the extracellular matrix. Panel **a** show the increased number of adherent human CHQ myoblasts cells on laminin and fibronectin substrates, in the absence or presence of HGF, as a function of time (15 min; 30 min; 1 h; 2 h). Adhesion was not enhanced in the presence of HGF. **b** Representative images of primary CHQ myoblasts cultured on ECM-coated (LN-111 or FN) surfaces in the absence or presence of HGF visualized by phalloidin alexa-488 staining and analyzed by fluorescence microscopy. Data of three independent experiments of 15 min and 2 h (200×). (Magnification bar: 100 μm). BSA: Bovine serum albumin; GT: gelatin; LN: Laminin-111; FN: Fibronectin. Bars represent a mean ± SD. Results correspond to three independent experiments. ***p < 0.001 versus BSA

Higher numbers of myoblasts were found to adhere onto FN as well as LN substrates as compared to BSA at the time points analyzed (*p* < 0.001; Fig. 2a). Additionally, a significant increase in the spreading of these cells was observed in the presence of ECM proteins (*p* < 0.001; Additional file 2, Fig. 2b). It should be noted that HGF alone, or combined with LN-111 and FN, did not modify the numbers of cells bound to the substrate (Fig. 2a) and did not change cell circularity and spreading (Fig. 2b, Additional file 2).

### HGF enhances ECM-driven human myoblast migration

**Fig. 3:**
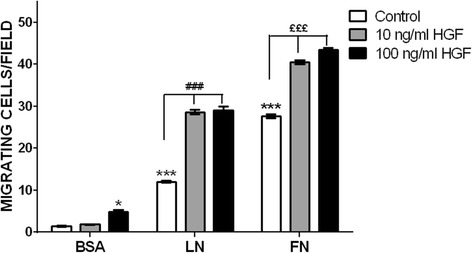
Effect of HGF treatment on the migration of human myogenic precursors (CHQ) towards ECM proteins. Hundred thousand human CHQ myoblasts were allowed to migrate across Transwell chambers, whose inserts were previously coated with BSA, laminin-111 (LN), or fibronectin (FN) plus HGF (10-100 ng/ml). The presence of HGF significantly enhanced the cell migration driven by LN and FN. Each bar represents the mean ± SE of four independent experiments. *p < 0.05 versus BSA; ***p < 0.001 versus BSA; ^###^ *p* < 0.001 versus LN; ^£££^ *p* < 0.001 versus FN

We investigated whether HGF could stimulate the migratory capacity of human myoblasts on ECM proteins using Transwell migration chambers. As expected, both LN-111 and FN induced specific myoblast haptotaxis as compared to the values obtained when cells were exposed to BSA, used as a negative control (Fig. 3; Additional file 3). Higher concentration of HGF (100 ng/ml) increased myoblast migration when compared to lower concentrations (10 ng/ml) or BSA control. When HGF was applied in combination with the ECM proteins, it enhanced the effect of LN and FN in CHQ cell migration. Interestingly, 10 ng/ml of HGF, despite not having a stimulatory effect on myoblast migration per se, synergistically stimulated myoblast migration when combined with the ECM proteins (Fig. 3; Additional file 3). Moreover, combined HGF + LN-111 increased migration approximately threefold as compared to LN-111 alone (control), while HGF + FN had a weaker, although significant, effect.

Since LN and FN accelerate adhesion, and at 2 h most of the cells presented a “spread” phenotype when attached on these substrates (Fig. 2b, Additional file 2), we decided to analyze cell migration in kinetics. Interestingly, the migratory capacity of human myoblasts on ECM molecules, combined or not with HGF, were, at 2 h, very similar to 4 h (Additional file 4).

One could ask if the non-myogenic cells present in the primary cell CHQ cultures (mainly fibroblasts) could play a role in the increased migration of CHQ cells observed under the influence of the ECM proteins and HGF. To answer this question, we repeated the same experiment with two immortalized cell clones, 100% myogenic; CL25, a clone derived from the CHQ cultures, and LHCN-M2 (a different donor). Both LN-111 and FN plus HGF induced an increased migratory capacity in both cell lines, similar to the migration of CHQ cells (Additional file 5).

### HGF enhances MMP secretion by human myoblasts: Interaction with the ECM-driven migratory response

**Fig. 4:**
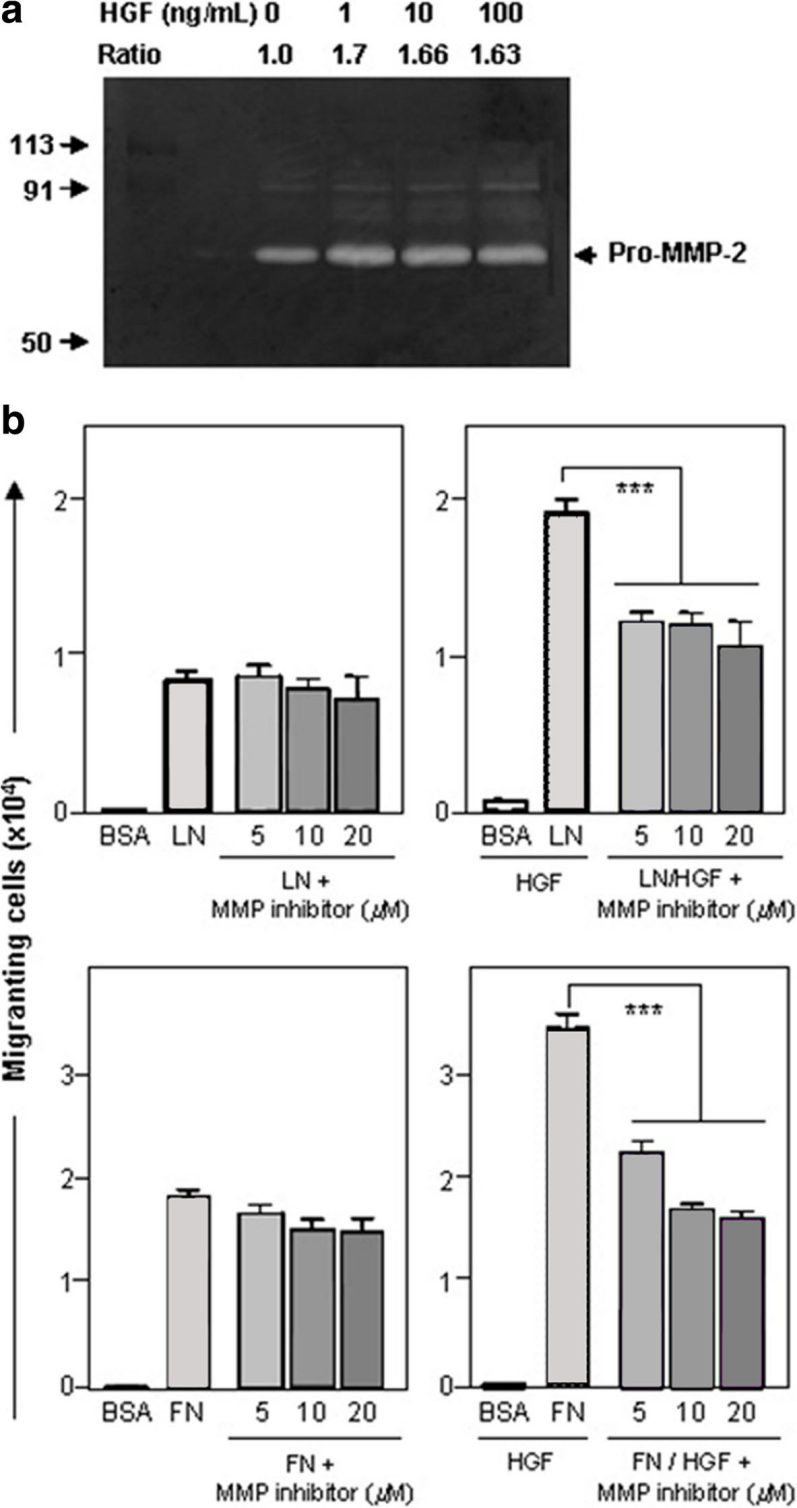
Matrix metalloproteinase production by human CHQ myoblasts: modulation of HGF-enhanced ECM-driven migration by MMP inhibition. **a** depicts MMP activity detected by gelatin zymography. Increasing doses of HGF (1 to 100 ng/mL) were added to CHQ myoblast cultures for 4 h. Supernatants were submitted to electrophoresis in gels co-polymerized with gelatin. The Pro-MMP-2 (72KDa) induced gelatin degradation, being visualized as clear bands. The ratio between treated and non-treated cultures was determined by band densitometry using ImageJ software. **b** Effect of MMP2/9 inhibitor on migration in the presence of laminin-111 (LN) or fibronectin (FN) without or with HGF. The MMP-2/MMP9 inhibitor significantly blocked the HGF-induced migration driven by both LN and FN. In this migration experiment, we detached the migrated cells with trypsin and counted the cells in a malassez chamber, giving the total number of migrated cells. Bars represent means ± SE from 3 independent experiments. ***p < 0.001

Since soluble HGF was able to regulate ECM-driven migration of human myoblasts, it seemed conceivable that HGF could induce the production of MMP-type enzymes by these cells. To test this hypothesis, human myoblasts were incubated with HGF for 72 h and the levels of MMP-2 and MMP-9 secreted into the culture medium were assayed by zymography. We found that treatment with HGF increased the activated MMP-2 levels, but had no effect on MMP-9 secretion (Fig. 4a).

To investigate whether the HGF-dependent increase in MMP-2 secretion could play a role in the migration of myogenic cells, we tested the effect of the selective MMP9/2 inhibitor decapeptide. When myoblasts were allowed to migrate through LN-111 or FN alone, no significant differences in the numbers of migrating cells were seen in the absence or the presence of the MMP2/9 inhibitor, applied at various doses. However, when HGF was added to the culture medium, the enhanced HGF + ECM-driven migration was significantly reduced (*p* < 0.05) by various concentrations of the inhibitor (Fig. 4b). Taken together, these data support a multistep process where HGF may induce MMP release, promoting HGF activation that would enhance cell migration by downstream effects.

### MAPK/ERK pathways are involved in the HGF-dependent migration induced by HGF

**Fig. 5:**
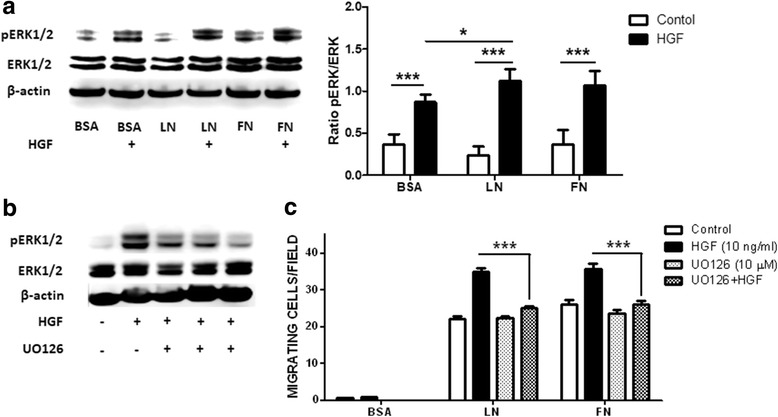
MAPK/ERK pathway modulation in the HGF-enhanced migration of human CHQ myoblasts. **a** Western blotting and densitometric analysis for ERK total (44–42 kDa) and p-ERK (44–42 kDa) in primary human CHQ myoblasts in the presence of laminin-111 (LN), fibronectin (FN), or BSA as control, and treated or not with HGF. MAPK/ERK is activated by HGF. Expression levels were normalized against the β-actin (~50 kDa) signal. Data are presented as mean ± SD. **b** Inhibition of p-ERK expression in human myoblasts co-treated with HGF and different concentrations of UO126 (2, 5, and 10 μM, right to left). **c** In the absence of HGF, the MAPK/ERK inhibitor does not modify myoblast migration towards LN-111 or FN. UO126 blocks the HGF-induced migration driven by both LN-111 and FN. Bars represent means ± SE from 4 independent experiments. *p < 0.05; ***p < 0.001

It is well known that HGF binding activates c-Met, which results in the phosphorylation of tyrosine residues, recruiting signaling effectors that activate mitogen-activated protein kinase (MAPK) and extracellular signal-regulated kinase (ERK), ultimately leading to cell migration [51, 52]. To investigate whether the enhancing effects induced by HGF treatment upon ECM-driven migration are dependent on the MAPK/ERK pathways, human myoblasts (CHQ cells) were incubated with medium alone or with 10 ng/ml HGF in pre-coated plates with BSA, FN, or LN-111. Lysates for SDS-PAGE were collected 15 min post-stimulation. We noticed that phospho-MAPK and phosphor-ERK were significantly increased in the cells treated with HGF, regardless whether myoblasts were plated on LN-111, FN, or BSA (*p* < 0.001; Fig. 5a). To further analyze the potential involvement of MAPK/ERK signaling in the enhanced migration of human myoblasts induced by HGF, cells were treated with different concentrations of UO126, a MAPK/ERK pathway inhibitor [53]. This treatment led to a decreased expression of phospho-ERK (Fig. 5b). While migration was not affected when myoblasts were treated with UO126 alone, the MAPK/ERK pathway inhibition reduced the synergistic effects of HGF upon migration induced by both LM-111 and FN (Fig. 5c). These results suggest that MAPK/ERK signaling is required for the enhanced ECM-driven myoblast migration following HGF treatment. These results, initially obtained with CHQ primary myoblasts, were also observed with the two immortalized lineages of human myoblasts (Additional file 5). These findings indicate that myoblasts from different donors (LHCN-M2 × CHQ/CL25 cells) respond in a very similar way to LN-111, FN, and HGF. The presence of contaminating cells, like fibroblasts, does not influence the migration of the myogenic CHQ cells, since the immortalized CL25 cell line (100% myogenic) also responded to these factors similarly as CHQ cells.

## Discussion

Myoblast migration is an essential step during muscle embryogenesis and muscle regeneration. Yet, it still represents one bottle-neck in myoblast transplant therapy, an alternative for the treatment of muscular dystrophies, which has given variable clinical benefits for patients depending on the disease [45, 54]. The movement to reach another myoblast or a damaged fiber, to fuse or to regenerate muscle, including potential association of activated and/or proliferating satellite cells creating oriented doublets which can have an influence on their fate, as suggested by Siegel et al. [55], occurs in the context of an extracellular milieu rich in soluble factors and ECM proteins [6, 7]. The role of Eph/ephrin in this context has been suggested by Stark et al. [56], as well as that of CD34 since CD34 defective murine satellite cells display a decreased motility [57]. HGF is also involved in the migration of different cell types, including myoblasts [36, 58–60]. This molecule acts via the specific receptor, c-Met, the met tyrosine kinase, being able to regulate skeletal muscle precursor cell migration during development [61, 62]. In adult muscles, HGF is the first signal that activates quiescent SC after trauma or disease, thus initiating muscle repair [63]. It also regulates proliferation and differentiation in both mouse and human myoblasts [29, 63, 64].

Laminin is a major basal membrane component of the muscle fiber, including the satellite cell niche [65]. Mutations in the LN α2 chain, which is part of the α2β1γ1 trimer (LN-211), the major LN isoform on muscle, cause severe muscular dystrophy [66]. Although LN-111 is not present in the normal adult muscle, it is the most studied LN isoform, also applied as a therapeutic agent in different animal models for muscular dystrophies, such as Duchenne muscular dystrophy (DMD) and merosin-deficient congenital muscular dystrophy type 1A (MDC1A) [22, 67–69]. Different LN isoforms can have differential effects on myoblast proliferation or differentiation, as shown recently for LN-211 or LN-521 [24]. Fibronectin is present in small quantity in adult muscle fibers and absent in SC niche [7, 70]. However, after muscle damage, FN is transiently expressed during regeneration being essential for SC expansion [71]. The ECM molecules not only stimulate the cells directly via their cell membrane receptors but can also play a role as haptotactic substrates [72]. Furthermore, ECM proteins can capture, store, and present soluble factors to a given cell [7, 73]. Both ECM molecules and growth factors are important elements in muscle environment during regeneration, although their combinatorial effect on myoblast motility remains poorly described. In our culture conditions, CHQ human myoblast myogenicity is higher than 75%; they express desmin and can differentiate in vitro, thus revealing their commitment to the muscle lineage. These myoblasts, as the immortalized cell lines, constitutively and simultaneously express the HGF receptor (c-Met), as well as integrin chains, such as CD49d, CD49e, CD49f, CD49g, and CD29, thus pointing to the presence of the integrins α4β1 and α5β1 (FN receptors), as well as α6β1 and α7β1 (LN receptors). Similar integrin expression was observed in primary and immortalized mouse cell lines [74].

As adhesion is involved in the migration process, we investigated the effect of HGF, LN, and FN on the adhesiveness of the human progenitor cells. Both ECM molecules accelerate adhesion and spreading of human myoblasts when compared to controls, BSA, and gelatin. Accordingly, the increasing adhesion and spreading induced by LN-111 and FN could accelerate myoblast migration and, consequently, have a physiological role in the regeneration process. HGF alone or combined with FN and LN-111 did not change the parameters promoted by the ECM proteins, indicating that HGF is not involved in myoblast adhesion. We confirmed previous data showing that LN and FN stimulate human myoblast migration, at least in vitro data [19, 20]. Moreover, we showed that HGF, although not having a migratory effect alone at a low dose, was able to enhance ECM-driven migration of human myoblasts. Different from 10 ng/ml, HGF treatment at the higher dose of 100 ng/ml, slightly but significantly, increased migration when compared to the control. Since both low and high concentrations enhanced migration promoted by LN and FN, we can use 10 ng/ml as an optimal concentration to further stimulate myoblast migration on ECM molecules. This is further supported by the data showing that rat and mouse myoblasts migrated more efficiently at low concentrations of HGF, ranging from 1 to 10 ng/ml, whereas the dose of 100 ng/ml decreased murine myoblast migration [58, 75].

Siegel et al. [74] published a report in which they show on murine isolated fibers, which have their basal lamina preserved, that although murine satellite cells proliferate and migrate in vitro on the native LN which surrounds these fibers, the addition of exogenous HGF did not increase myoblast velocity. In this system, where migration is analyzed on longer periods, the fibers could be already saturated by HGF already produced and/or stored before addition of exogenous HGF. Conversely, they observed that HGF increased directional persistence and decreased tortuosity. In contrast to these in vitro experiments using isolated fibers, the addition of HGF in the bottom well of the Transwell migration chambers will form a gradient, stimulating chemoattraction and increasing migration, but only in the presence of a proper substrate, such as LN-111 and FN. It was demonstrated that murine myoblasts and myotubes, but not fibroblasts, produce HGF, which acts autocrinally, but its mRNA is detected only 12 h after plating [76]. These results probably exclude myoblast-secreted HGF in our short-course migration assay. Moreover, c-Met mutant cells had a perturbed lamellipodia formation and decreased velocity [29]. The human myoblasts used in our study produce LN and FN in culture, and additional production or deposition of these proteins could modulate myoblasts migration. However, no increase of LN and FN production was observed up to 4 h of culture after plating, even in the presence of HGF (data not shown).

We believe that such an interplay between ECM and HGF can modulate human myoblast migration onto ECM proteins. Crosstalk between receptors for growth factors and ECM molecules, i.e., integrins, have been described as part of the mechanical sensing machine of the cell [77–79]. The synergic response between HGF with LN and FN could be a consequence of a cross-talk between c-Met and β1-integrins [80]. It has been suggested that β1-integrins could mediate migration, and c-Met mutant myoblasts, which lack elaborated lamellipodia and had a lower frequency of peri-nuclear β1-integrin localization, present a decreased motility [29]. Furthermore, HGF can enhance human endothelial cell migration by a mechanism involving association between c-Met and integrins, through the Ras-activation pathway [73]. Indeed, c-Met binding to the integrin α6β4 (another integrin-type LN receptor) creates a supplementary docking platform for signaling molecules, thus amplifying Met signal [81, 82]. We showed herein that HGF synergizes with LN-111 and FN to stimulate human myoblast migration, indicating that the oriented movement of human myogenic precursors may follow a complex pattern of response to distinct simultaneous and/or sequential stimuli, including the possible involvement of MMPs.

We investigated a putative MMP modulation by HGF and as part of ECM-mediated biological responses. Previous studies showed that HGF induces the migration of myoblasts from mouse tongue by modulating MMP-9 expression [83]. We showed that human myoblasts cultured with different concentrations of HGF presented an increased MMP-2 gelatinolytic activity, possibly related to the enhancement of in vitro ECM-driven migration. Importantly, in the absence of HGF, MMP-2 blockade with a specific MMP-2/9 inhibitor did not result in any significant changes in cell migration driven by LN-111 or FN, whereas in the presence of HGF plus ECM there was a partial but significant decrease in the number of migrating cells.

Although the improvement of myoblast engraftment has been observed after muscle MMP-1 treatment [84], the actual mechanisms through which the MMPs modulate myoblast migration remain to be defined. Substrate proteolysis with consequent exposure of cryptic adhesion sites to ECM might be involved; however, the addition of an MMP inhibitor alone (i.e. in the absence of HGF) had no significant effect on migration driven by LN-111 or FN.

We observed that HGF increased ERK1/2 phosphorylation in human myoblasts. Interestingly, HGF-stimulated human myoblasts produced more pERK1/2 phosphorylation in the presence of LN than with BSA. This result suggests that LN-111 can increase HGF-induced pERK1/2 phosphorylation. The involvement of the ERK/MAPK pathway was further confirmed using a specific inhibitor of this pathway. Accordingly, it seems that the MAPK/ERK signaling is required for the enhancing effect of HGF. One possible mechanism could involve an increased expression or activation of integrins on the membrane. This is supported by the data showing that HGF increases adhesion of multiple myeloma cells on FN by activating the MAPK/ERK pathway, also dependent on VLA-4 expression [85, 86].

Also, LN-111 induces integrin expression on myoblasts and has been used to improve muscle regeneration in different animal models of muscular dystrophy [22]. In this context, it is very reasonable that the positive effect of muscle repair after LN injection could also be a consequence of a better migratory capacity of muscle progenitor cells, which in turn, are imbibed with local growth factors.

## Conclusions

In conclusion, we have demonstrated that HGF alone applied in relatively low dose does not improve human myoblast migration per se, but can play an important pro-migratory role when associated with ECM proteins. This effect is likely related to MMP-2 secretion induced in these cells and the activation of the MAPK/ERK pathway. Taken together, these data provide new clues to help us understand the complexity of the control mechanisms involved in the migration of human myoblasts, simultaneously involving soluble and insoluble moieties. These findings will help to optimize the design of future cell therapy strategies to treat muscle damage and disease, improving cell migration and muscle formation within the recipient’s tissue by the effect of trophic factors.

## Additional files

Additional file 1: A) depicts the mean fluorescence intensities (MFI) derived from cytofluorometry for detection of CD49d, CD49e, and CD49f integrin alpha-chains from myoblast cell lines. B) shows cytofluorometric histograms for CD56 immunodetection in CL25 and LHCN-M2 human myoblast clones. (TIFF 245 kb)

Additional file 2: Kinetic analysis showing the spread area and cellular circularity of human myoblasts (CHQ cells) adhered onto ECM protiens, with or without HGF. **p < 0.01 versus BSA; ***p < 0.001. *GT* gelatin; *LN* laminin-111; *FN* fibronectin. (TIFF 181 kb)

Additional file 3: Migrated human myoblasts onto laminin-111 and fibronectin coated membranes in combination or not with soluble HGF (10–100 ng/ml). Cell stained with Panotic kit. (Magnification bar: 100 μm). (TIFF 964 kb)

Additional file 4: A) Kinetic of the human myoblast migration through 8-μm pore filters, pre-coated with the laminin-111 (LN) and fibronectin (FN), in the presence or not of 10 ng/ml HGF in the lower chamber. B) Cells stained with Panotic kit after 30 min of migration (Magnification bar: 100 μm). *p < 0.001 versus BSA; ^#^ *p* < 0.01 versus LN; ^£^ *p* < 0.05 versus FN. (TIFF 672 kb)

Additional file 5: Migration of human myoblast cell lines towards laminin-111 (LN) or Fibronectin (FN), in the presence or absence of HGF, with ou without it inhibitor (UO126). Graph bars represent means error standard, showing migration of LHCN-M2 (10^5^ cells) and CL25 (5 × 10^4^ cells) human myoblasts onto extracellular matrix protiens in the presence or not of 10 ng/ml HGF. Note that UO126 alone does not have any effect on cell migration, but it does block the enhancing effects of HGF upon LN- or FN-induced migration. ***p < 0.001. (TIFF 79 kb)

## Abbreviations

BSA: Bovine serum albumin
ECM: Extracellular matrix
FN: Fibronectin
HGF: Hepatocyte growth factor
LN: Laminin
MMP: Matrix metalloproteinase
MRF: Myogenic Regulatory Factors
SC: Satellite cell
