# Supplementary figures and images for: HGF potentiates extracellular matrix-driven migration of human myoblasts: involvement of matrix metalloproteinases and MAPK/ERK pathway
Source: Skelet Muscle. 2017 Oct 10;7:20. doi: 10.1186/s13395-017-0138-6 (PMC5635537; doi:10.1186/s13395-017-0138-6)

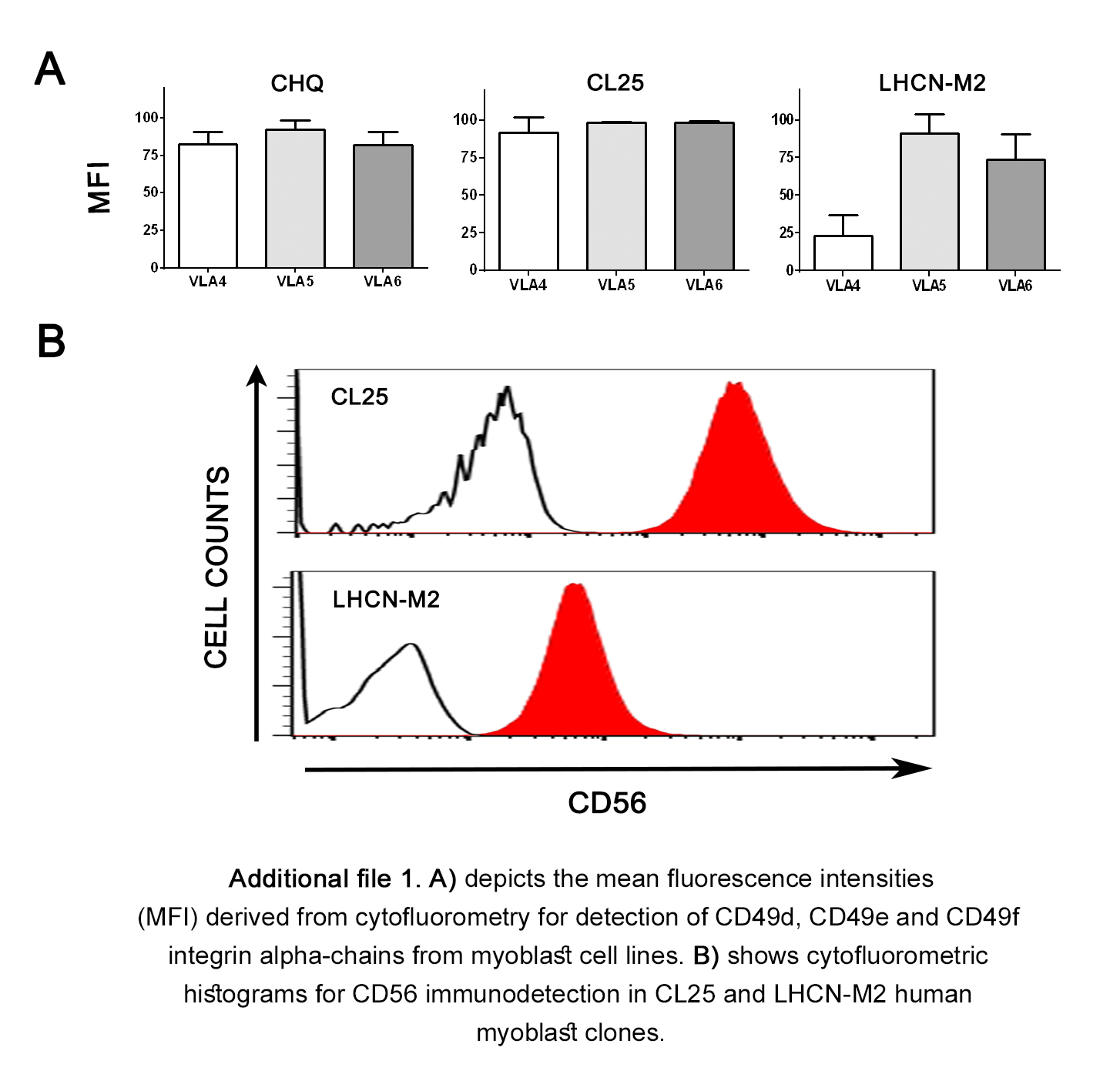

Supplement: Supplementary file 1 — A) depicts the mean fluorescence intensities (MFI) derived from cytofluorometry for detection of CD49d, CD49e, and CD49f integrin alpha-chains from myoblast cell lines. B) shows cytofluorometric histograms for CD56 immunodetection in CL25 and LHCN-M2 human myoblast clones. (TIFF 245 kb) [file 13395_2017_138_MOESM1_ESM.tif]

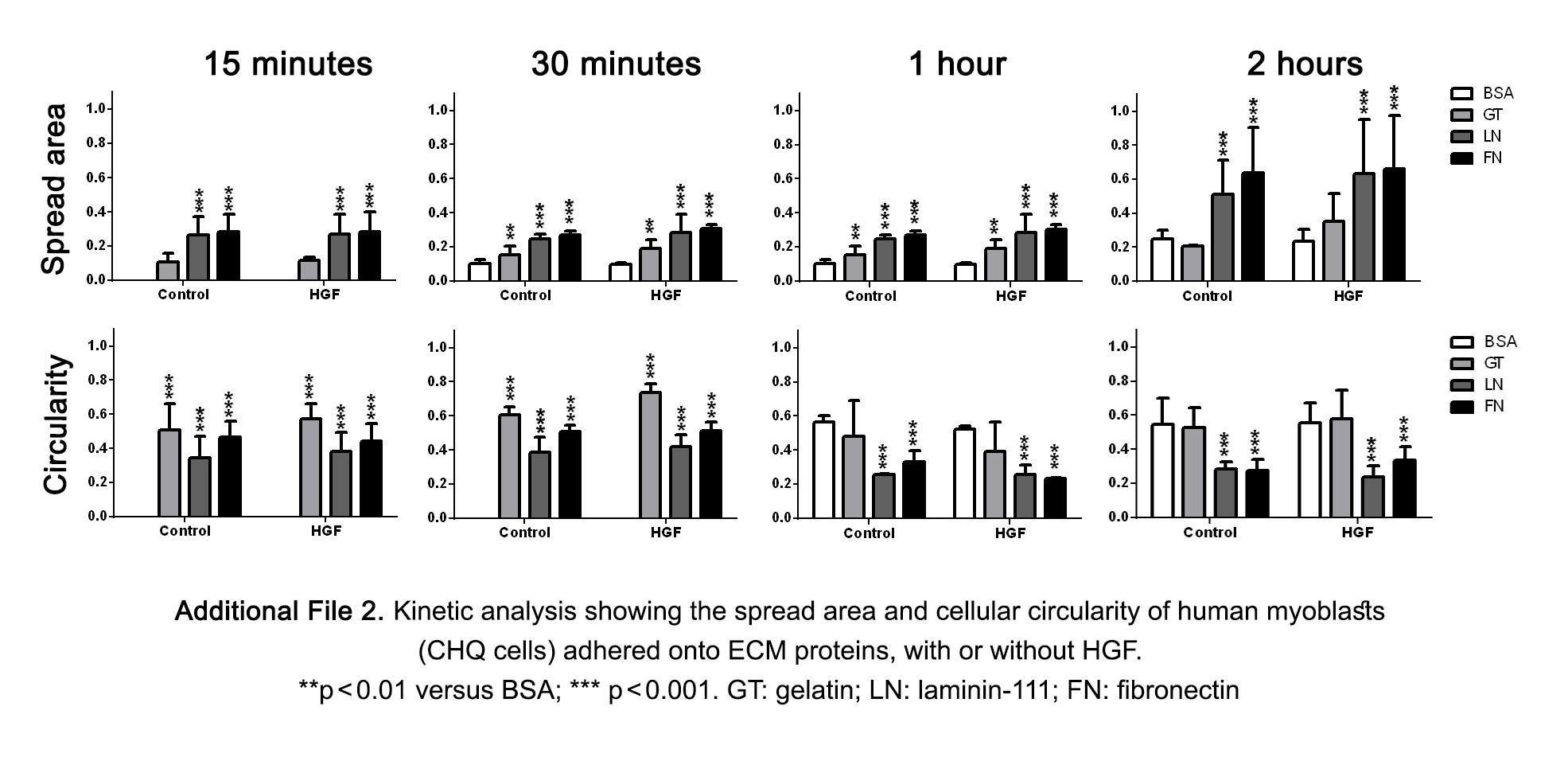

Supplement: Supplementary file 2 — Kinetic analysis showing the spread area and cellular circularity of human myoblasts (CHQ cells) adhered onto ECM protiens, with or without HGF. **p < 0.01 versus BSA; ***p < 0.001. GT gelatin; LN laminin-111; FN fibronectin. (TIFF 181 kb) [file 13395_2017_138_MOESM2_ESM.tif]

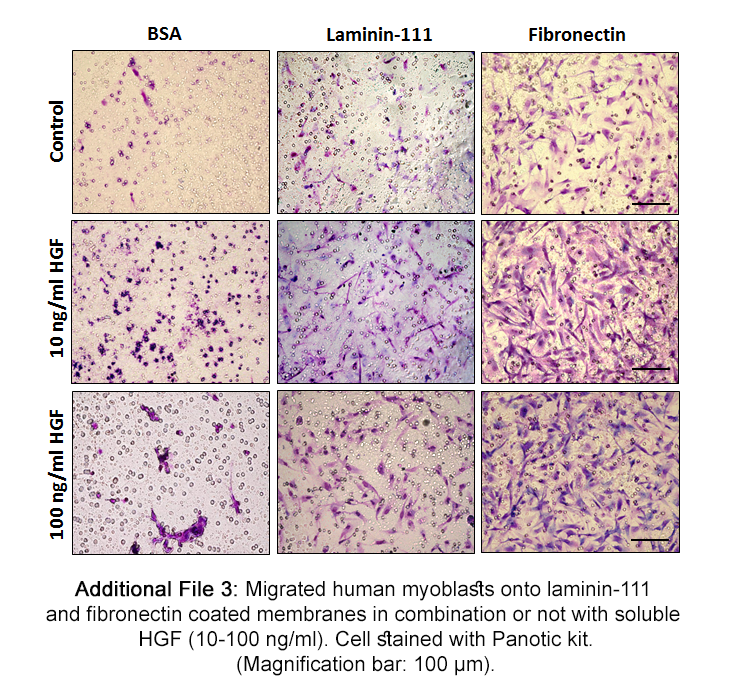

Supplement: Supplementary file 3 — Migrated human myoblasts onto laminin-111 and fibronectin coated membranes in combination or not with soluble HGF (10–100 ng/ml). Cell stained with Panotic kit. (Magnification bar: 100 μm). (TIFF 964 kb) [file 13395_2017_138_MOESM3_ESM.tif]

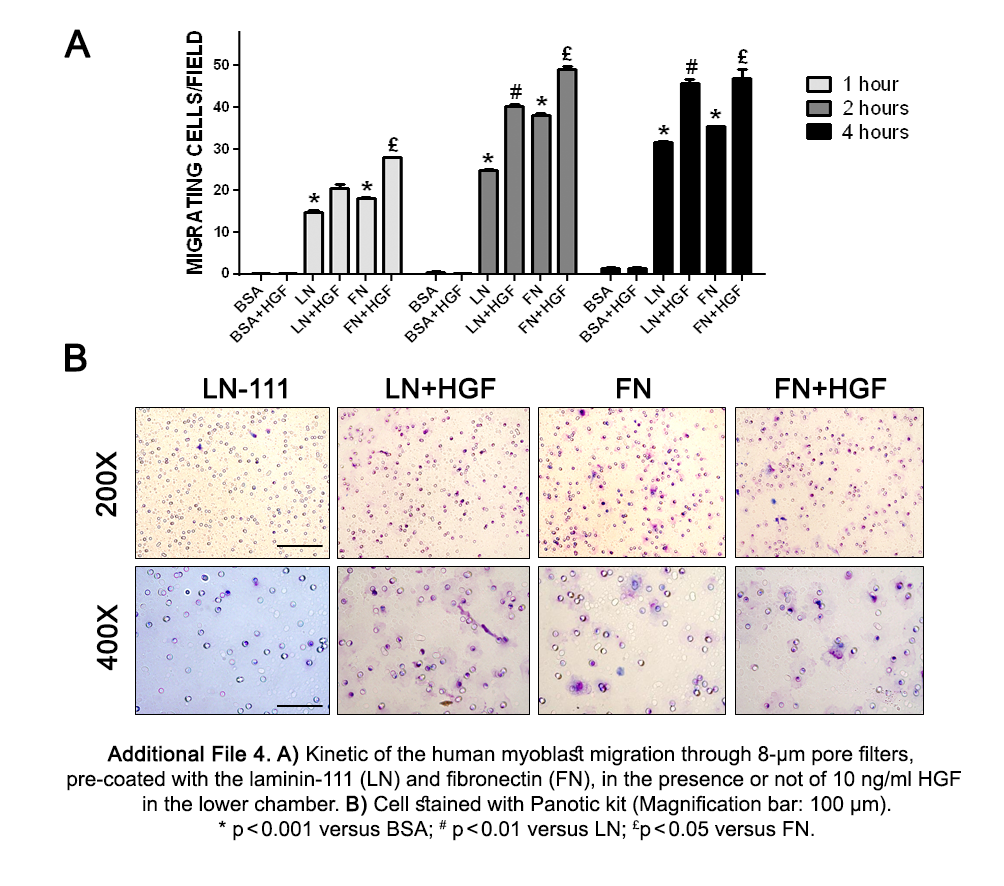

Supplement: Supplementary file 4 — A) Kinetic of the human myoblast migration through 8-μm pore filters, pre-coated with the laminin-111 (LN) and fibronectin (FN), in the presence or not of 10 ng/ml HGF in the lower chamber. B) Cells stained with Panotic kit after 30 min of migration (Magnification bar: 100 μm). *p < 0.001 versus BSA; # p < 0.01 versus LN; £ p < 0.05 versus FN. (TIFF 672 kb) [file 13395_2017_138_MOESM4_ESM.tif]

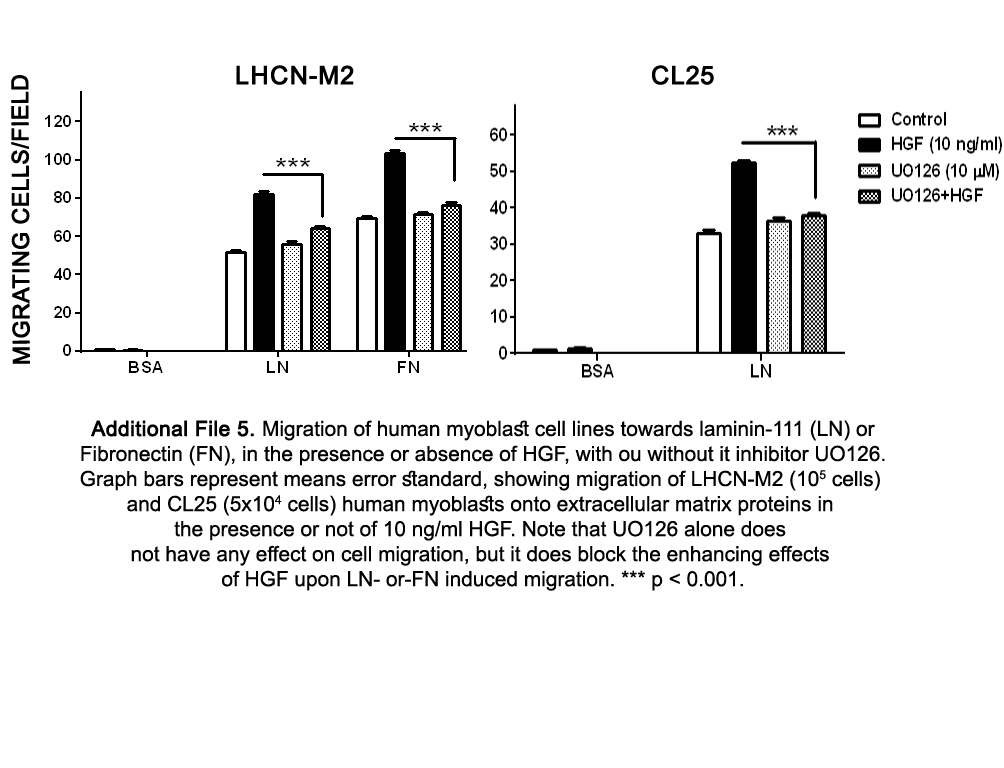

Supplement: Supplementary file 5 — Migration of human myoblast cell lines towards laminin-111 (LN) or Fibronectin (FN), in the presence or absence of HGF, with ou without it inhibitor (UO126). Graph bars represent means error standard, showing migration of LHCN-M2 (105 cells) and CL25 (5 × 10^4^ cells) human myoblasts onto extracellular matrix protiens in the presence or not of 10 ng/ml HGF. Note that UO126 alone does not have any effect on cell migration, but it does block the enhancing effects of HGF upon LN- or FN-induced migration. ***p < 0.001. (TIFF 79 kb) [file 13395_2017_138_MOESM5_ESM.tif]
